# Phosphorus Dendrimers as Carriers of siRNA—Characterisation of Dendriplexes

**DOI:** 10.3390/molecules18044451

**Published:** 2013-04-15

**Authors:** Malgorzata Ferenc, Elzbieta Pedziwiatr-Werbicka, Katarzyna E. Nowak, Barbara Klajnert, Jean-Pierre Majoral, Maria Bryszewska

**Affiliations:** 1 Department of General Biophysics, Faculty of Biology and Environmental Protection, University of Lodz, 141/143 Pomorska St., Lodz 90-236, Poland; 2 Division of Radiobiology, Department of Molecular Biophysics, Faculty of Biology and Environmental Protection, University of Lodz, 141/143 Pomorska St., Lodz 90-236, Poland; 3 Laboratoire de Chimie de Coordination du CNRS, 205 route de Narbonne, 31077 Toulouse Cedex 4, France

**Keywords:** siRNA, phosphorus dendrimers, chronic myeloid leukemia, gene therapy, dendriplex

## Abstract

There are many types of dendrimers used as nanomolecules for gene delivery but there is still an ongoing search for ones that are able to effectively deliver drugs to cells. The possibility of gene silencing using siRNA gives hope for effective treatment of numerous diseases. The aim of this work was to investigate *in vitro* biophysical properties of dendriplexes formed by siRNA and cationic phosphorus dendrimers of 3rd and 4th generation. First, using the ethidium bromide intercalation method, it was examined whether dendrimers have an ability to form complexes with siRNA. Next, the characterisation of dendriplexes formed at different molar ratios was carried out using biophysical methods. The effects of zeta potential, size and changes of siRNA conformation on the complexation with dendrimers were examined. It was found that both phosphorus dendrimers interacted with siRNA. The zeta potential values of dendriplexes ranged from negative to positive and the hydrodynamic diameter depended on the number of dendrimer molecules in the complex. Furthermore, using circular dichroism spectroscopy it was found that cationic phosphorus dendrimers changed only slightly the shape of siRNA CD spectra, thus they did not induce significant changes in the nucleic acid secondary structure during complex formation.

## 1. Introduction

Despite great efforts, vast resources and impressive progress in medicine and biology in recent years, scientists still have failed to find effective drugs for the treatment of people suffering from leukemia, and there is a constant search for new and improved pharmaceuticals for this disease.

Gene therapy has received significant attention due to its potential use in replacement of abnormal genes and treatment of so far incurable diseases [1,2]. Today, researchers’ hopes are set on gene therapy, which involves the introduction of fragments of nucleic acids into the cell to silence the gene. Small interfering siRNAs (siRNA) are a new class of drugs for cancer. These small molecules mediate a process called RNA interference, in which expression of a homologous gene is silenced or inhibited [3]. In recent years there is a growing interest in siRNAs because of their specificity and efficiency [4].

The problem which gene therapies encounter is drug delivery to cells in an efficient, easy and the least toxic way. As soon as nanosystems were developed to carry drugs into the cells, many research teams have used these technologies to create nanosystems for effective siRNA delivery. The use of new, synthetic nanoparticles as carriers of nucleic acids may prove helpful in delivery. One of these promising drug transporters are dendrimers. Some of these monodisperse polymers were synthesized and described by Tomalia in 80s of the last century [5]. With many advantages, such as the uniform shape and size, the presence of free voids within their backbone and numerous functional groups on the surface, dendrimers can efficiently bind the drug and serve as its carrier [6]. The precise structure of the dendrimers and the ability to tailor them will make them the best, as far, potential drug carriers.

In recent years several studies have been undertaken on the potential use of phosphorus dendrimers [7,8] in medicine. Most of the potential applications of these dendrimers in biology are connected with their water-solubility [9,10]. Because of their hydrophilic surface and hydrophobic backbone phosphorus dendrimers are able to penetrate membranes [11,12]. It has recently been reported that phosphorus dendrimers can interact with model membranes. Moreover these dendrimers affected the phase behaviour of lipid vehicles and the physical properties such as surface charge of membranes [13,14]. These results show that phosphorus dendrimers can be helpful to solve some problems of drug delivery.

Polyanionic phosphorus dendrimers bearing bisphosphonic end groups were found to dramatically induce the amplification of NK cells, first line of our immune system, and to activate monocytes via an anti-inflammatory way [15,16,17,18].

One of our previous papers describes interactions between cationic phosphorus dendrimers of generation 4 (pdG4, see Table 1) and anti-HIV P24 siRNA. The characterisation of formed complexes using different methods like circular dichroism, fluorescence polarization, zeta potential and hydrodynamic diameter shows that pdG4 can bind anti-HIV siRNA [19]. Moreover, these results allow us to speculate that phosphorus dendrimers are good candidates to become effective carries for drugs in anti-HIV therapy.

In the present work we describe the properties of complexes formed by phosphorus dendrimers of generation 3 and 4 with siRNAs directed against the BCR gene responsible for the development of chronic myeloid leukemia.

**Table 1:** Stern–Volmer constants for quenching of ethidium bromide bound to siRNA fluorescence by phosphorus dendrimers.

****	**K_sv_ [L/mol]**	**V [L/mol]**
pdG3	1.1 × 10^6^	1.5 × 10^6^
pdG4	2.1 × 10^6^	2.9 × 10^6^

## 2. Results and Discussion

### 2.1. Ethidium Bromide Intercalation Assay

The ability of cationic phosphorus dendrimers of generation 3 and 4 to form complexes with siRNA was assessed by observing changes in the shape and intensity of the EB fluorescence emission spectrum in the presence of siRNA after adding dendrimers. 

**Figure 1:**
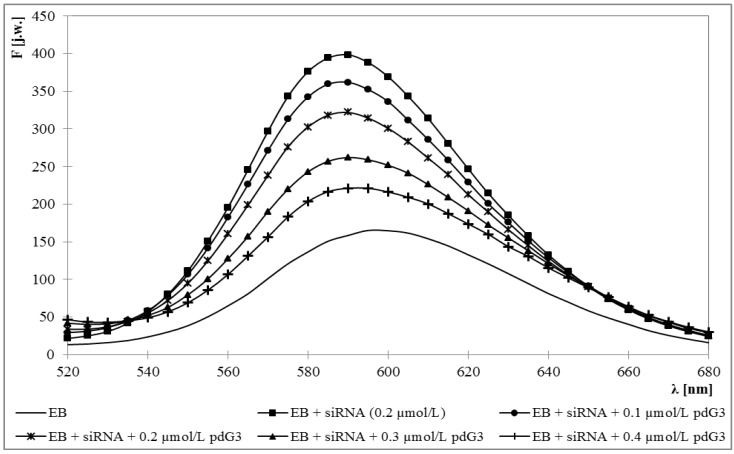
Fluorescence spectrum of ethidium bromide in the presence of siRNA and increasing concentrations of phosphorus dendrimer generation 3 (pdG3). [EB] = 5 μmol/L [siRNA] = 0.2 µmol/L.

**Figure 2:**
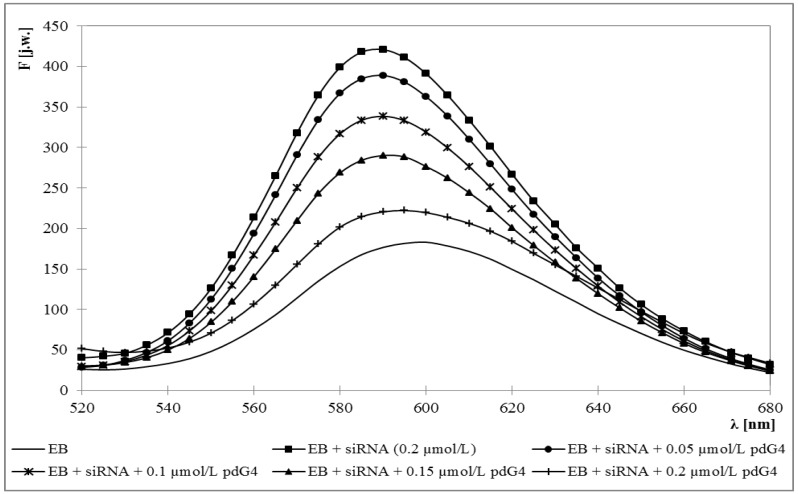
Fluorescence spectrum of ethidium bromide in the presence of siRNA and increasing concentrations of phosphorus dendrimer generation 4 (pdG4). [EB] = 5 μmol/L [siRNA] = 0.2 µmol/L.

Fluorescence emission maximum for ethidium bromide was obtained at 595 nm. Upon addition of siRNA this maximum became blue-shifted to 588 nm and approximately 3-fold increase in fluorescence intensity was observed. Adding the dendrimers caused the decrease of fluorescence intensity and a back shift toward longer wavelengths to 595 nm (Figure 1 and Figure 2).

**Figure 3:**
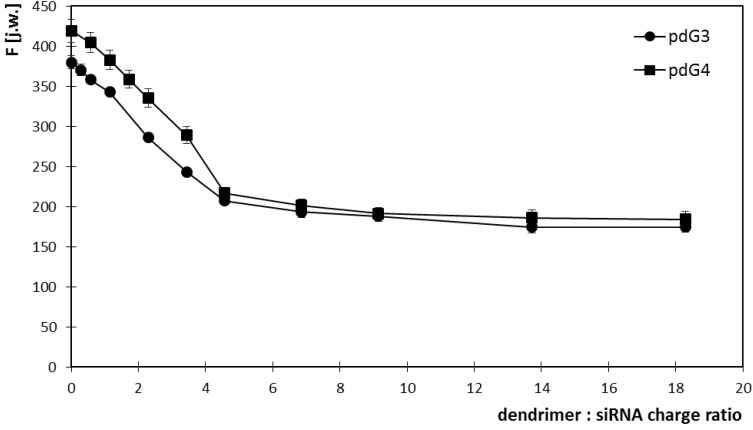
Changes in the intensity of ethidium bromide fluorescence vs the concentration of phosphorus dendrimers generation 3 (pdG3) and 4 (pdG4) at 588 nm. [EB] = 5 μmol/L [siRNA] = 0.2 µmol/L.

Increasing concentrations of dendrimers allowed for determination of molar ratios in which they form dendriplexes with siRNA. For this purpose, the graphs of EB fluorescence intensity at 588 nm *versus* dendrimer concentrations were drawn (Figure 3). The concentration of siRNA in the samples was constant and amounted to 0.2 μmol/L. 

The graphs show that phosphorus dendrimers of generation 3 formed complexes with siRNA in the molar ratio 2:1 (charge ratio 5:1), which means that one molecule of siRNA can bind two molecules of pdG3. For pdG4 dendrimers molar ratio is 1:1—one siRNA molecule can bind one molecule of pdG4 (charge ratio 5:1). Concentration of dendrimers in such complexes was adequately 0.4 μmol/L for pdG3 and 0.2 μmol/L for pdG4.

Because the quenching of EB fluorescence was observed upon addition of dendrimers, the Stern-Volmer graphs were drawn. Typical Stern–Volmer equation for the dynamic quenching has the form:

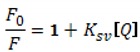
(1)

In our experiments, the Stern-Volmer plots for both dendrimers were not linear for the tested quencher (dendrimer) concentrations. The presence of an upward-curving Stern-Volmer plots indicates the presence of the static quenching mechanism which affects the fluorescence according to:

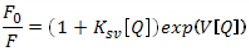
(2)
where F is fluorescence intensity in presence of dendrimer, F_0_—fluorescence intensity in absence of dendrimer, [Q]—the dendrimer concentration, K_SV_—the overall quenching constant, V—the static quenching constant [20].

**Figure 4:**
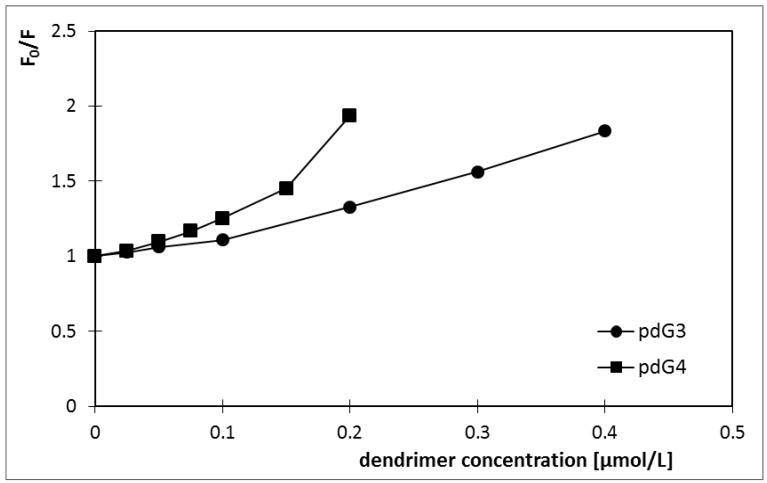
Stern-Volmer plots for ethidium bromide fluorescence quenching by phosphorus dendrimers generation 3 (pdG3) and 4 (pdG4).

In the first step of analysis, the linear dependence for [Q] ≤ 0.2 μmol/L for pdG3 and [Q] ≤ 0.1 μmol/L for pdG4 was observed (Figure 4) (taking into consideration the correlation confidence—R2 > 0.99), so after fitting it with Equation (1) the values of K_SV_ were determined. The K_SV_ values of 1.1 × 10^6^ L/mol for pdG3 and 2.1 × 10^6^ L/mol for pdG4 were obtained.

For higher [Q] concentrations (above 0.2 and 0.1 μmol/L for pdG3 and pdG4, respectively) the upward-curving of Stern-Volmer plots was observed. So in the next step, we used the obtained K_SV_ values to fit the data to Equation (2) and estimate the V value. The Stern-Volmer constants obtained are given in Table 1.

### 2.2. Zeta Potential

**Figure 5:**
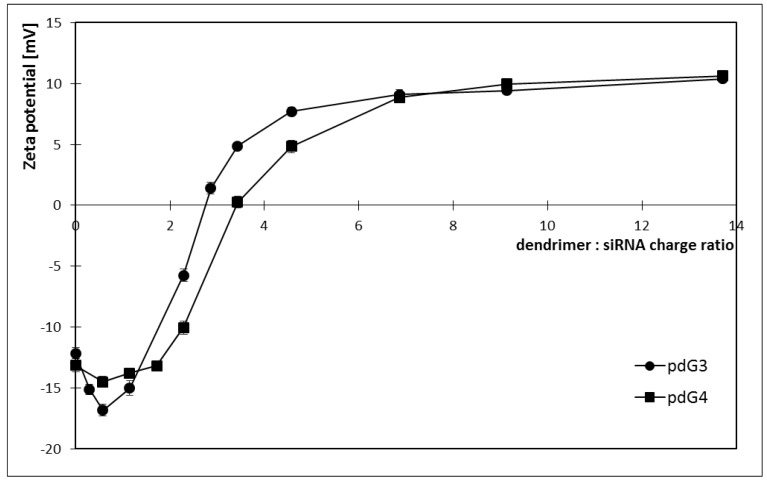
Changes in the zeta potential of dendriplexes formed by siRNA phosphorus dendrimers generation 3 (pdG3) and 4 (pdG4) [siRNA] = 0.2 µmol/L.

Zeta potential of siRNA in the buffer was negative and amounted to about −12/−13 mV. After adding phosphorus dendrimers endowed with positive charge, initially a slight decrease in zeta potential and then its rise to the positive values was observed (Figure 5).

Phosphorus dendrimers of generation 4 have more cationic groups on the surface than phosphorus dendrimers of generation 3, therefore complexes siRNA:pdG4 have higher values of zeta potential for the same molar ratios than complexes siRNA: pdG3.

From these graphs, the charge ratios in which dendriplexes are formed can be obtained. For pdG3 phosphorus dendrimers this ratio was 5:1 (molar ratio 2:1), while for the generation 4 the charge ratio was 7:1 (molar ratio 1.5:1). The zeta potential values for these molar ratios are +7.7 mV for pdG3 and +8.9 mV for pdG4. For pdG3 the zeta potential is equal to 0 for dendrimer: siRNA charge ratio 1:3 while for pdG4 it equals to 0 mV for the charge ratio 1:3.

### 2.3. Hydrodynamic Diameter

**Figure 6:**
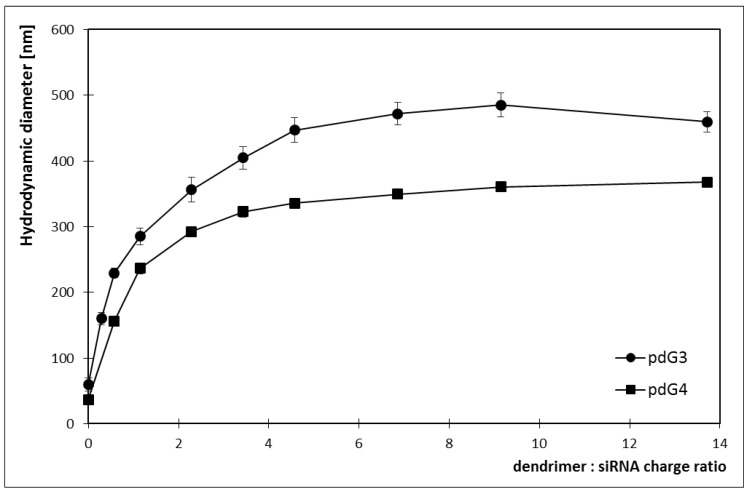
Changes in the hydrodynamic diameter of dendriplexes formed by siRNA phosphorus dendrimers generation 3 (pdG3) and 4 (pdG4) [siRNA] = 0.2 µmol/L.

Using dynamic light scattering the hydrodynamic diameter of complexes was determined. With increasing concentration of dendrimer in the sample the size of dendriplexes was increasing reaching the plateau for a certain siRNA: dendrimer molar ratio (Figure 6). The value of the hydrodynamic diameter increased to the point where it was impossible to attach more dendrimers to siRNA strand.

By measuring the size of the complexes, it was possible to evaluate charge ratios in which dendriplexes are created. For the generation 3 dendrimer this ratio was 5:1 (molar ratio 1:1) and hydrodynamic diameter of a complex was about 445 nm. Phosphorus dendrimers of generation 4 formed complexes with siRNA at a molar ratio of 1:1 (charge ratio 5:1), and the size of a complex was approximately 335 nm. For the same molar ratios pdG3 dendrimers formed larger complexes with siRNA than pdG4 dendrimers. 

### 2.4. Circular Dichroism

Detection of the CD spectra for different siRNA concentrations allowed for the selection of a concentration, for which the signal was strong enough to observe changes in the spectra shape at adding dendrimers to the sample. It was important that the signal significantly exceeded the noise level. For this reason, for further experiments, siRNA concentration equal to 2 μmol/L was selected.

**Figure 7:**
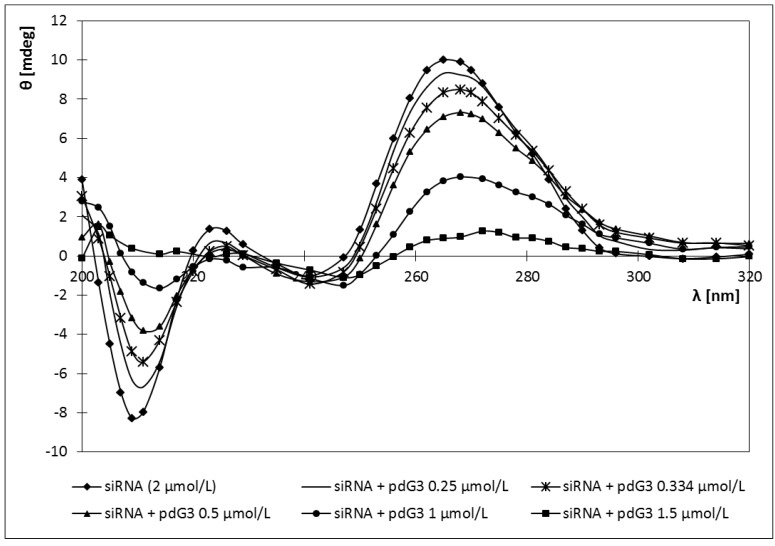
CD spectra of siRNA in the presence of different concentrations of phosphorus dendrimers generation 3 (pdG3) [siRNA] = 2 µmol/L.

**Figure 8:**
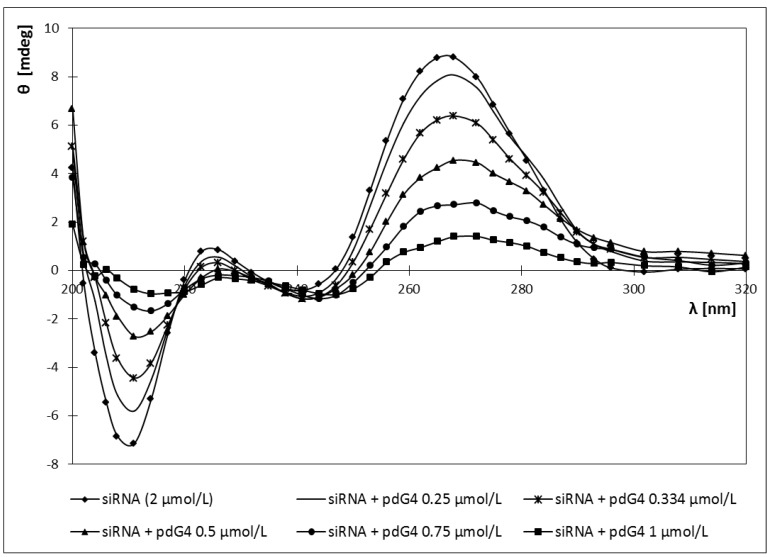
CD spectra of siRNA in the presence of different concentrations of phosphorus dendrimers generation 4 (pdG4) [siRNA] = 2 µmol/L.

Adding both cationic phosphorus dendrimers to the sample of siRNA caused a red shift of the CD spectra maximum from 265 nm (for pure siRNA) to 272 nm (Figure 7 and Figure 8).

**Figure 9:**
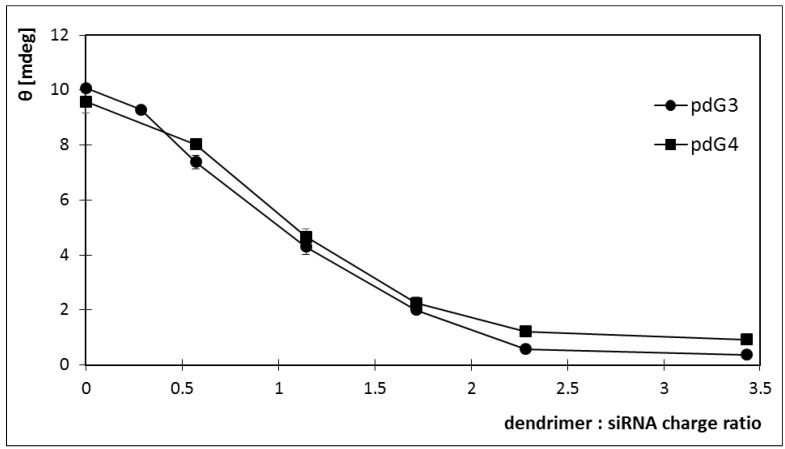
Changes in the ellipticity of dendriplexes formed by siRNA phosphorus dendrimers generation 3 (pdG3) and 4 (pdG4) [siRNA] = 2 µmol/L, λ = 265 nm.

Also siRNA ellipticity decreased with increasing concentrations of the dendrimers. For both dendrimers ellipticity reached a value close to 0 (Figure 9). For pdG3 dendrimers this value was reached at a concentration of 1.5 μmol/L, while for pdG4 dendrimers at a concentration of 1 μmol/L. Decreasing the molar ellipticity sharply to zero upon addition of pdG3 and pdG4 dendrimers indicates the formation of complexes between siRNA and phosphorus dendrimers. 

### 2.5. Discussion

Finding an effective drug delivery system to cells, its characteristics and subsequent use in gene therapy is the key to the use of siRNA for the treatment of cancer. Since naked nucleic acids or oligodeoxynucleotides (ODNs) are poorly transported into cells by endocytosis [21,22], a delivery system is required for antisense activity in cell culture. Studies confirm that ODN:dendrimer complexes can enter cells by adsorptive endocytosis more effectively than the naked oligodeoxy-nucleotides [23].

Scientists and biotechnology companies have developed many vectors for delivery of siRNA into the cytoplasm of target cells, and although they are sufficient for most applications *in vitro*, not all are suitable for use *in vivo* [24]. Currently in clinical trials, siRNA is directly delivered to local destinations, such as eyes, lungs or respiratory system (e.g., in an aerosol form). In this way the complexity of delivery systems is avoided [25,26].

The optimal system for delivering siRNA *in vivo* must have the following characteristics. First, the vector should be biocompatible, biodegradable and non-immunogenic. Secondly, it is necessary for the carrier to provide an effective delivery of siRNA to target cells and also to protect the double-stranded siRNA against nuclease attack found in serum. Then, the specificity for tissues distribution in the body and the possibility of immediate transfer of the vector–drug complex to the liver or kidneys must be taken into account. Finally, after delivery to target cells via endocytosis, the system should release the siRNA into the cytoplasm, which allows for forming RISC complex and initiating the RNA interference that leads to the corresponding gene silencing [27,28]. Despite the vast number of publications about different types of dendrimers, phosphorus dendrimers appear among the most promising dendrimers for gene delivery.

In this work characterization of complexes formed by siRNA and phosphorus dendrimers was made in order to determine the usability of these dendrimers as carriers of siRNA in gene therapy directed against chronic myeloid leukemia. 

It was possible to determine whether siRNAs form complexes with phosphorus dendrimers of generation 3 and 4 and in what molar ratios, and to characterize formed dendriplexes using biophysical methods. Due to their cationic surface groups, dendrimers can interact with negatively charged phosphate groups of siRNA. Creation of dendriplexes is based on electrostatic interactions between these groups [29].

Efficiency of creating dendriplexes depends on environmental conditions such as pH or temperature. All experiments performed in this study were carried out as close as possible to physiological conditions: at a temperature of 37 °C and pH 7.4.

The use of ethidium bromide intercalation allowed to show that phosphorus dendrimers of both generations are able to form complexes with tested siRNA either at a siRNA:dendrimer ratio 1:2 (pdG3) or 1:1 (pdG4). The lower generation pdG3 dendrimer is smaller, has a lower molecular weight, so the greater number of its molecules can attach to siRNA. The values of the ratios at which siRNA can form complexes with dendrimers were next confirmed by Laser Doppler Electrophoresis and Dynamic Light Scattering measurements. 

The phosphorus dendrimer of generation 4 pdG4, which possesses two times more terminal amino groups than dendrimer of generation 3 [10], quenched the fluorescence of ethidium bromide stronger than pdG3 (Figure 3 and Figure 4). The Stern-Volmer constants were higher for pdG4 which means a better availability of ethidium bromide bound to the siRNA to the quencher (dendrimer). Upward curvature of the Stern-Volmer plots is often noticed for efficient quenchers and is due to the formation of non-fluorescent complex between the fluorescent molecule and a quencher. It may be accompanied by changes in the structure of molecule [30]. 

Circular dichroism is a sensitive but qualitative indicator of changes in RNA conformation [31,32]. Detection of the CD spectra for siRNA and siRNA:dendrimer complexes was designed to determine whether cationic phosphorus dendrimers affect the conformation of short interfering nucleic acids. 

In our study the CD spectra of the siRNA showed the characteristic A-form pattern [33], with a strong positive peak at ~265 nm and a negative peak at ~210 nm (Figure 7 and Figure 8). Addition dendrimers to the sample did not cause any changes in the shape of the spectrum, only a shift towards longer waves (265→272) nm for positive peak and (210→217) nm for negative peak (Figure 7 and Figure 8). It can thus be concluded that the cationic phosphorus dendrimers of generations 3 and 4 do not alter significantly the secondary structure of siRNA and a decrease in ellipticity is a proof of a complex formation.

Zeta potential measurements of complexes showed that with increasing dendrimer concentration (number of molecules) in the sample the value of the zeta potential also increased. For both generations of phosphorus dendrimers zeta potential of dendriplexes reached positive values. It was equal to +7.7 mV for the phosphorus dendrimer of generation 3 and +8.9 mV for the fourth-generation-dendrimer (Figure 5). Positive charge of complexes facilitates the transport of drug into the cell because the cell membranes are negatively charged and thus the complex will not be repelled by them. On one hand the use of complexes with such a high zeta potential protects them from aggregation but on the other hand, it may lead to activation of complement proteins and removal of dendriplexes by the immune system and may also be the cause of low stability in biological fluids *in vivo* [34]. *In vitro* a positive charge is desired, as it increases the solubility of the complexes in the aqueous environment [35]. Hence, there is the need to use complexes with a value of zeta potential slightly higher than zero. Similar experiments were carried out on complexation of siRNA directed against luciferase with PAMAM dendrimers of generation 1, 4 and 7 [36], however the dendriplexes which were formed for various molar ratios were either highly negative (PAMAM G1, approx. −30 mV) or highly positive (G4 and G7, approx. +40 mV). It shows that PAMAM dendrimers cannot be considered to be good candidates for siRNA delivery because they form dendriplexes which will either be repelled from negatively charged biomembranes or will induce immunological response.

One of the obstacles to transport siRNA using phosphorus dendrimers can be the size of dendriplexes. Hydrodynamic diameter measured by DLS method was approximately 445 nm for the siRNA: pdG3 dendriplexes, while for the complexes of siRNA:pdG4 it equalled to 335 nm (Figure 6). Dendriplexes of this size may be too large to freely pass through the pores in the membrane of tumor cells whose cytoplasm is the target of siRNA. Such large complexes are due to aggregation of dendrimers, which is a result of electrostatic interactions and hydrogen bonds [37].

Very similar results were obtained in studies on complexes formed by pdG4 and anti-HIV p24 siRNA. In these studies, upon addition of pdG4, the molar ellipticity decreased sharply to zero and big changes in zeta potential of siP24 were also observed. Moreover, the hydrodynamic diameter of siP24-pdG4 complexes did not exceed 500 nm [14]. All these results are quite similar to those obtained in the present study. The only difference was the molar ratio, at which complexes were formed, but this may be due to the different sequence of the nucleic acid. Nevertheless, both studies show that phosphorus dendrimers of generation 4 easily bind different types of siRNAs and a characterization of these complexes shows that they may be potential drug carriers for anticancer and anti-HIV gene therapy.

It seems that the supply of siRNA into cells is easier than DNA, because in order to induce a therapeutic effect it is sufficient that the siRNA reaches the target cell cytoplasm (where the phenomenon of RNA interference occurs), in contrast to DNA, which must be delivered to the nucleus to induce gene silencing [38].

The length of short interfering ribonucleic acids is also significant. The first studies on RNA interference phenomenon have used siRNAs with a length of more than 30 base pairs. It turned out that these molecules induced the expression of interferon, which resulted in inhibition of translation and degradation of all mRNA molecules in the cell. Thanks to the work of Tuschl and his team it was demonstrated that a synthetic 21-nucleotide RNAs attenuate target genes, without the induction of interferon expression [4]. In our studies we used siRNAs with a length of 21 base pairs, therefore eliminating the above effect.

## 3. Experimental

### 3.1. Materials

Phosphorus dendrimers generations 3 and 4 were synthesized in the Laboratoire de Chimie de Coordination du CNRS in Toulouse (France) [9]. Both dendrimers used in this study have pheripheral cationic groups. The characterisation of dendrimers is presented in Table 2. 

**Table 2:** Characterisation of phosphorus dendrimers of generation 3 and 4.

**Dendrimer**	**MW (Da)**	**Structure**	**Number of cationic end groups**
**pdG3**	16280	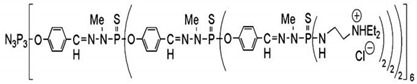	48
**pdG4**	33702	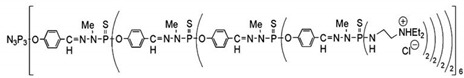	96

siRNA used in the study is directed against BCR gene which is responsible for the development of chronic myeloid leukemia (CML). This siRNA was purchased from Applied Biosystems and its characterisation is presented in Table 3.

**Table 3:** Characterisation of siRNA.

****	**Sense**	**Antisense**
Sequence (5'→3')	CGCAUGUUCCGGGACAAAAtt	UUUUGUCCCGGAACAUGCGgt
Length (number of base pairs)	21	21
G/C content	48%	48%
Molar weight	6800	6600
Total molar weight	13,400	

Ethidium bromide (EB) was obtained from Sigma-Aldrich (St. Louis, MO, USA). Double-distilled water was used to prepare all solutions. In this study 0.15 mol/L phosphate buffer (pH 7.4) was used.

### 3.2. Ethidium Bromide Intercalation Assay

Ethidium bromide (EB), a fluorescent dye, because of its chemical structure can intercalate into a DNA and RNA strand [30]. The result of the dye intercalation is a blue-shifted and increased intensity of EB fluorescence [39].

EB fluorescence was excited at 480 nm and fluorescence emission spectra of ethidium bromide in the 500–800 nm region were recorded with a PerkinElmer LS 55 fluorimeter. The excitation and emission slit widths were set at 10 and 15 nm, respectively. All samples were contained in 1 cm path length quartz cuvettes and were continuously stirred. Before the fluorescent properties of siRNA-EB-dendrimer complexes were examined it was established that the dendrimers did not interact with EB.

Ethidium bromide and siRNA were used at final concentrations of 5 and 0.2 μmol/L, respectively, in 0.15 mol/L phosphate buffer, pH 7.4 (1 molecule of EB per 4 bp of siRNA). First, fluorescence spectra of free EB were recorded. The fluorescence of free EB has the emission maximum at 595 nm. Then siRNA was added and the spectrum of fluorescence was recorded. Next, the increasing amounts of dendrimers were added to the system. Emission spectra were measured 5 min after the addition of dendrimers.

The data are expressed as mean values ± SEM of seven independent experiments for both generations of dendrimers. Statistical significance was assessed using Shapiro-Wilk and t-Student tests.

### 3.3. Laser Doppler Electrophoresis (LDE)

Zeta potential of dendriplexes was measured by LDE. All measurements were performed using the Zetasizer Nano—ZS (Malvern Instruments Ltd., Malvern, UK) at 37 °C and in 0.15 mol/L phosphate buffer, pH = 7.4. Zeta potentials of pure siRNA and siRNA:dendrimer dendriplexes in different molar ratios after 5 min of incubation were examined. siRNA concentration was 0.2 μmol/L and dendrimers concentrations, the same as in EB intercalation assay, increased from 0.025 μmol/L to 1.2 μmol/L. For each sample, measurements were performed 5 times in triplicate. The analysis of the zeta potential changes for increasing concentrations of dendrimers provided information about the maximum number of molecules of pdG3/pdG4 that can be attached to a single siRNA molecule. Statistical significance was assessed as given above.

### 3.4. Dynamic Light Scattering (DLS).

Hydrodynamic diameters of particles were measured in 0.15 mol/L phosphate buffer, pH = 7.4 at 37 °C by dynamic light scattering using Zetasizer Nano—ZS from Malvern. DLS measures Brownian motions and relates this to the size of particles by illuminating the particles with a laser light (633 nm) and analysing the intensity fluctuations in scattered light.

Hydrodynamic diameter measurements were made for pure siRNA (0.2 μmol/L) and for complexes formed by siRNA with dendrimers in different molar ratios after 5 min incubation. For each sample measurements were performed four times in four replications. Statistical significance was assessed as given above.

### 3.5. Circular Dichroism (CD).

In order to obtain the CD spectra of sufficiently strong signal intensity it was necessary to choose the appropriate concentration of siRNA. For this purpose, CD spectra were plotted for different concentrations of siRNA. Due to the need to have a strong signal during the measurements it was impossible to apply the same low concentrations of both siRNA and dendrimers as in previous experiments. Graphs for too low concentrations may come from noise, so it seemed reasonable to use in further research siRNA concentration of 2 μmol/L.

Next, to the siRNA samples, increasing concentrations of dendrimers (pdG3/pdG4) were added, in order to verify whether phosphorus dendrimers affect secondary structure of siRNA, depending on their concentration and generation. 

The CD spectra were plotted in the wavelength range (200–320) nm in a cuvette with dimensions of 5 mm × 5 mm. Measurements were made in four replications for each generation of dendrimer on JASCO J-815 spectropolarimeter. Statistical significance was assessed using Shapiro-Wilk and t-Student tests.

## 4. Conclusions

Gene therapy using siRNA may prove more effective than other antisense technologies. An example is the use of siRNA molecules to silence DNA methyl transferases. Studies conducted with nucleotides and antisense ribozymes gave incomplete inhibition of expression and, consequently, did not cause sensitivity to drugs, while the use of siRNA can bring the expected results [40]. 

Do cationic phosphorus dendrimers have the potential to become efficient carriers of siRNA in gene therapy? Yes—if one chooses the appropriate molar ratios of siRNA:dendrimer, and thus the charge and size of complexes, so that dendrimers can fulfill all the requirements they face. There is a need for more studies that will fully address dendrimers properties, their toxicity and potential behaviour in human body. However, the cationic phosphorus dendrimers offer hope to be effectively used as siRNA carriers in anticancer therapies.
